# Supplementary material for: Photons for Photography: A First Diagnostic Approach to Polaroid Emulsion Transfer on Paper in Paolo Gioli’s Artworks
Source: Molecules. 2022 Oct 18;27(20):7023. doi: 10.3390/molecules27207023 (PMC9607993; doi:10.3390/molecules27207023)
Supplement: Supplementary file 1 [file molecules-27-07023-s001.zip › molecules-1859408-supplementary.pdf]

Supplementary Materials: Photons for Photography: A Multi-Spectroscopic Approach to Diagnostics of Polaroid Emulsion Transfer on Paper in Paolo Gioli's Artworks

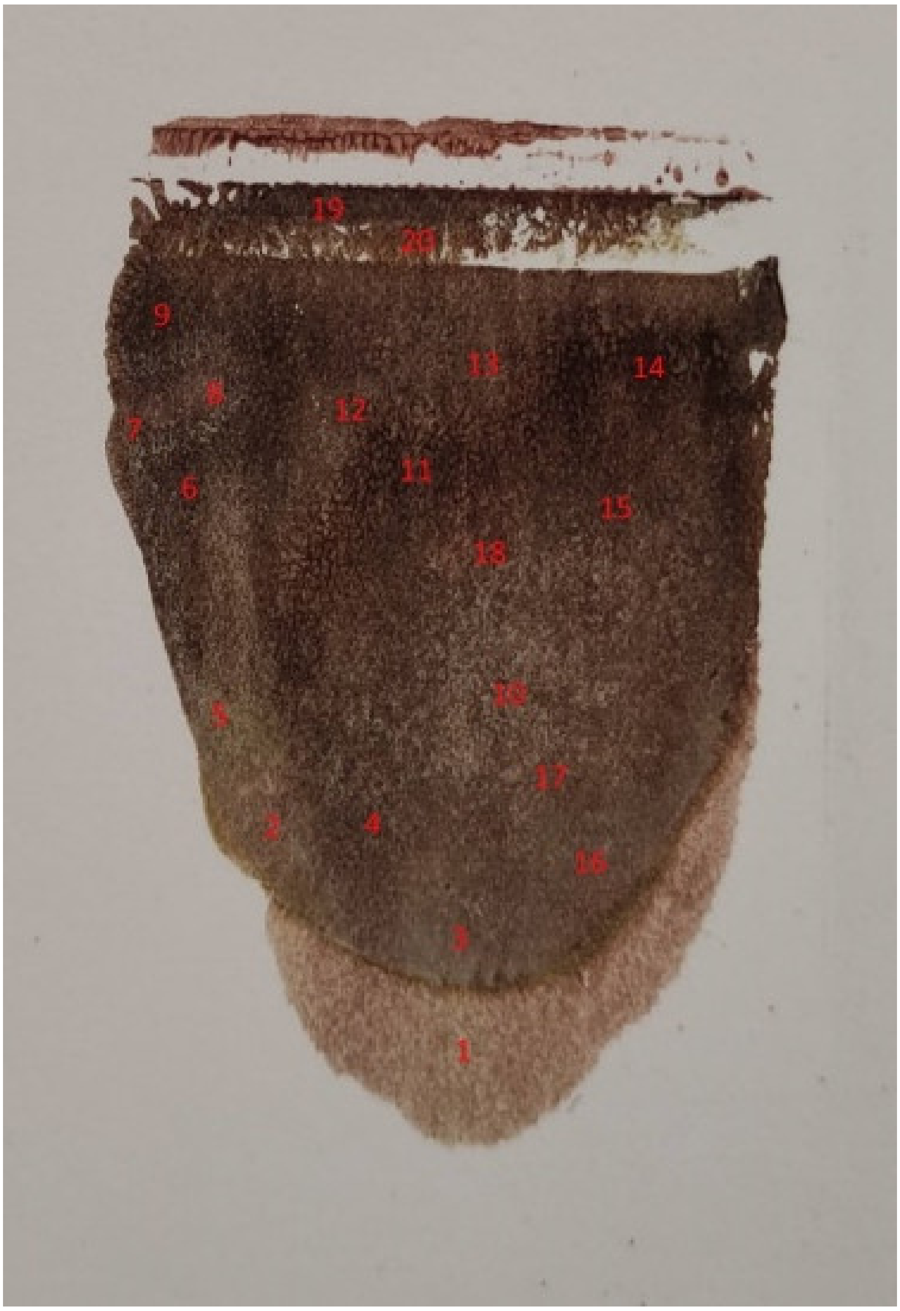

Figure S1: Points of the mock-up sample analysed by FORS spectroscopy.

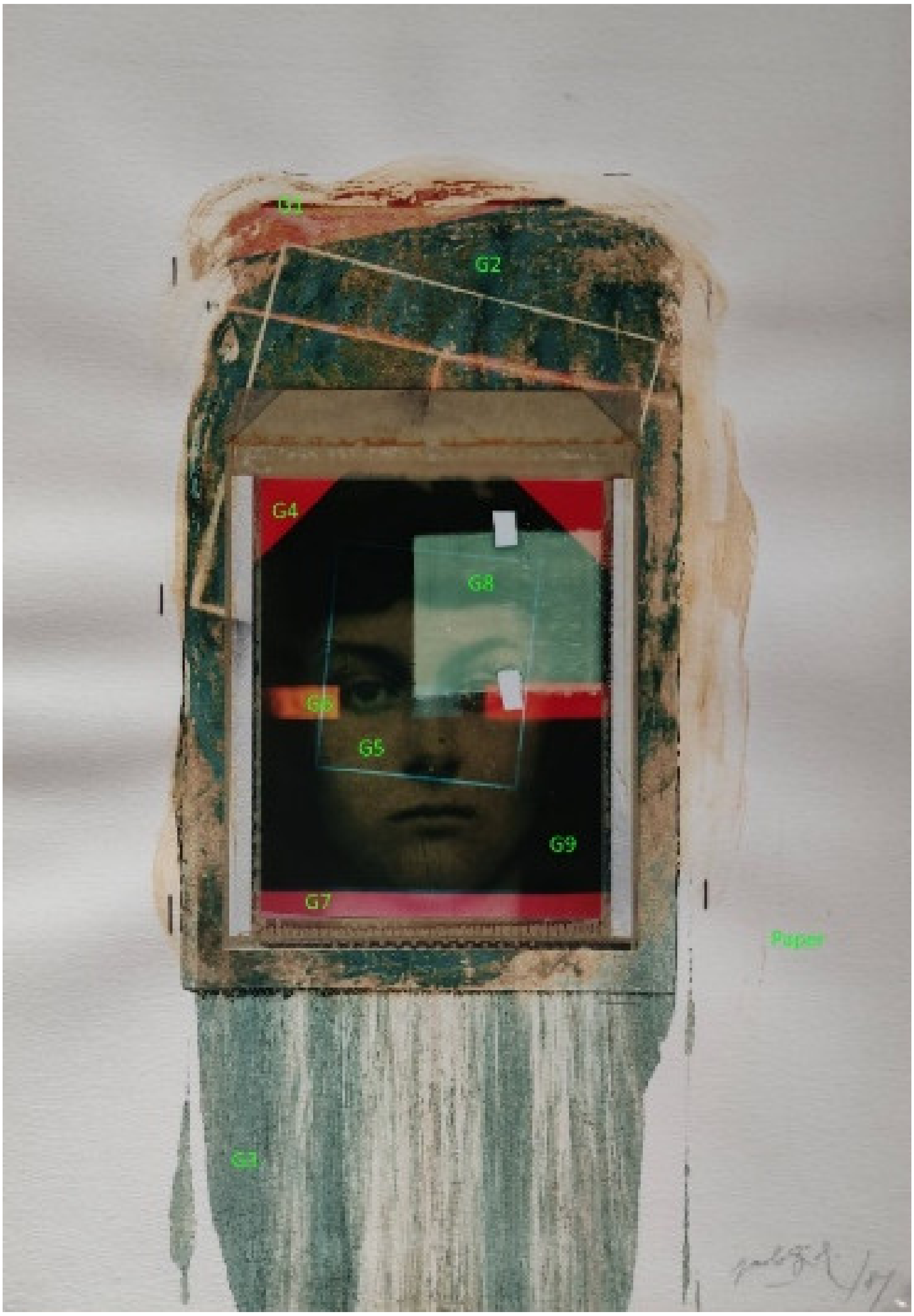

Figure S2: Points of Paolo Gioli’s artwork analysed by FORS spectroscopy.

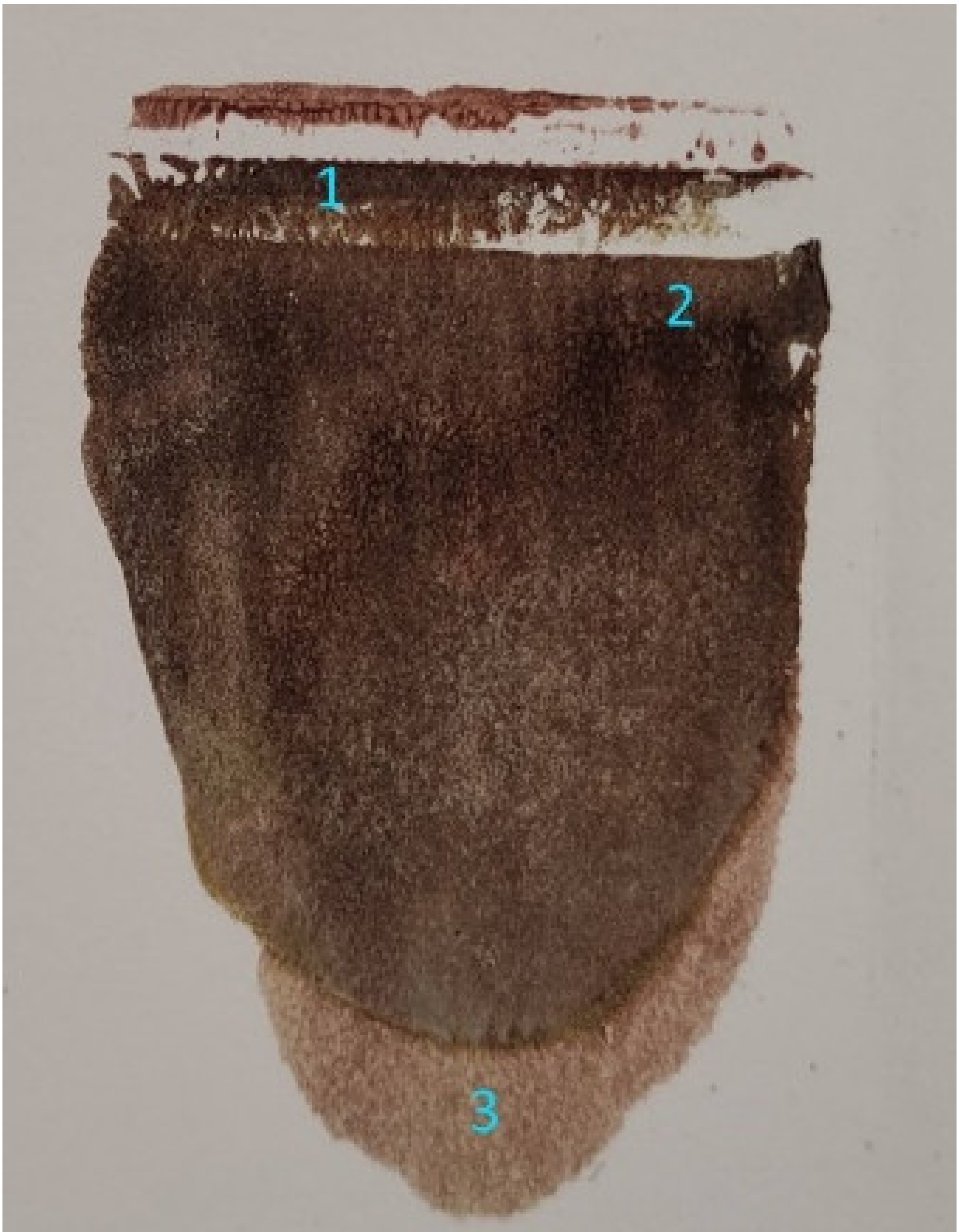

Figure S3: Points of the mock-up sample analysed by Raman spectroscopy.

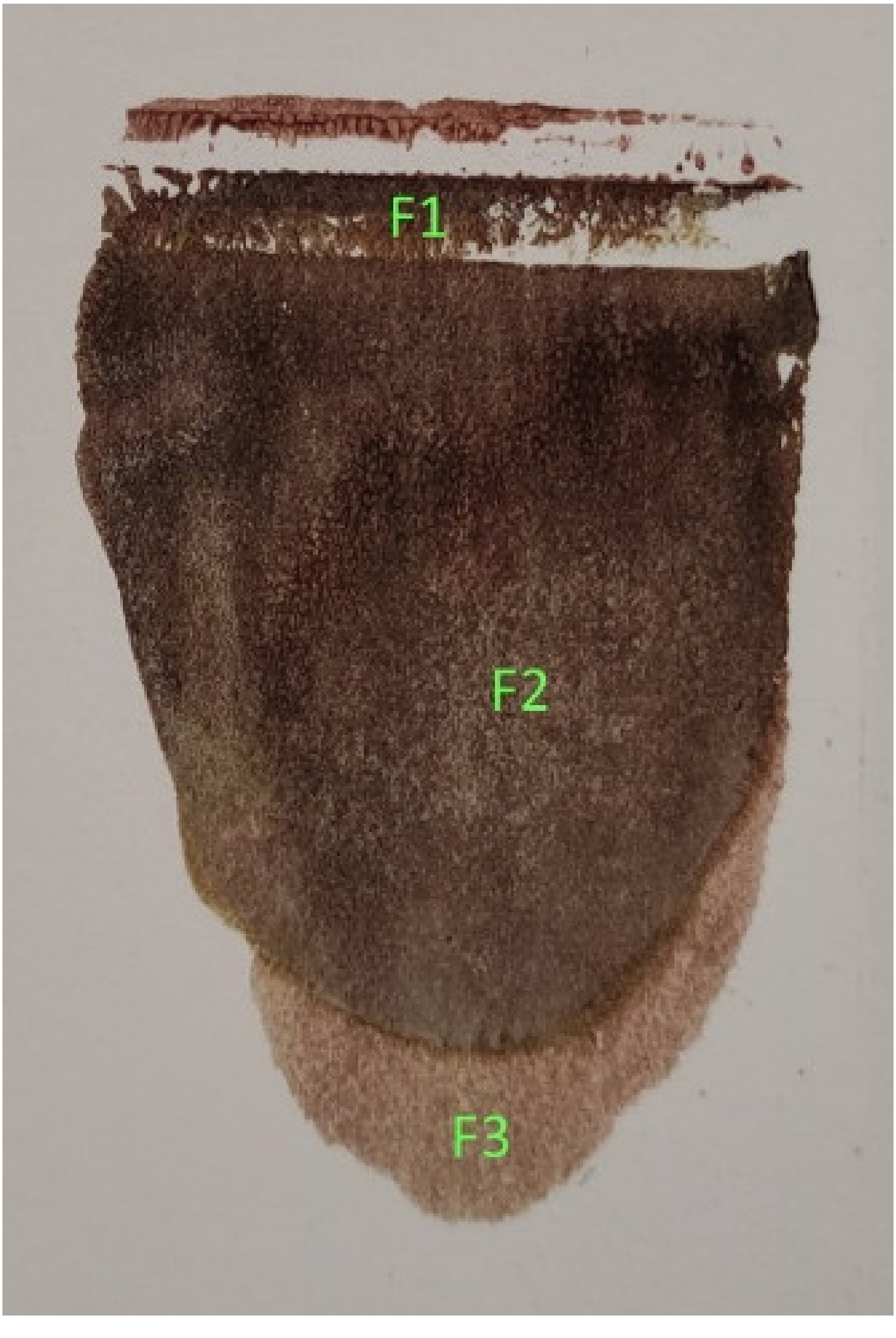

Figure S4: Points of the mock-up sample analysed by FTIR spectroscopy.

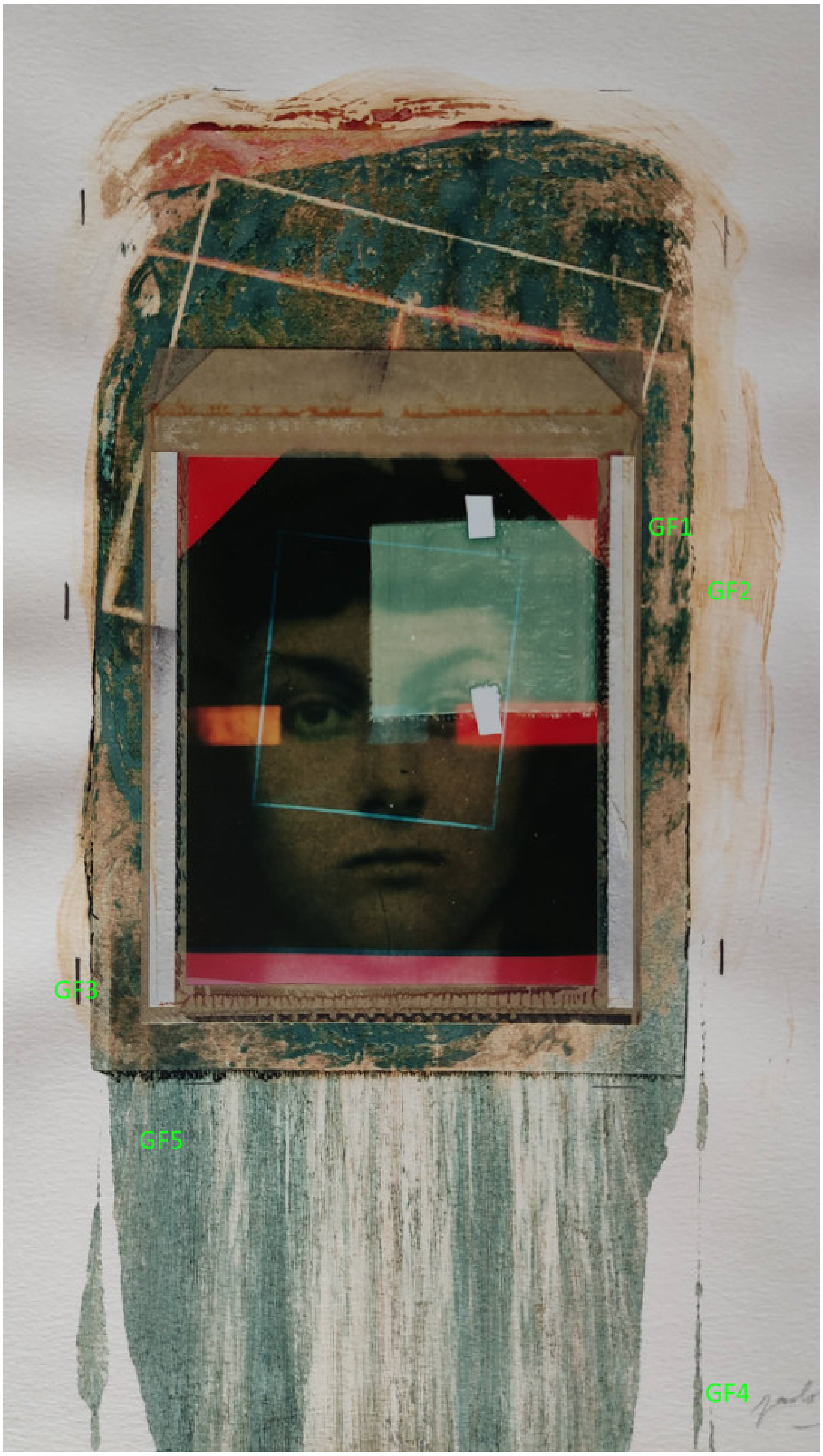

Figure S5: Points of Paolo Gioli's artwork analysed by FTIR spectroscopy.

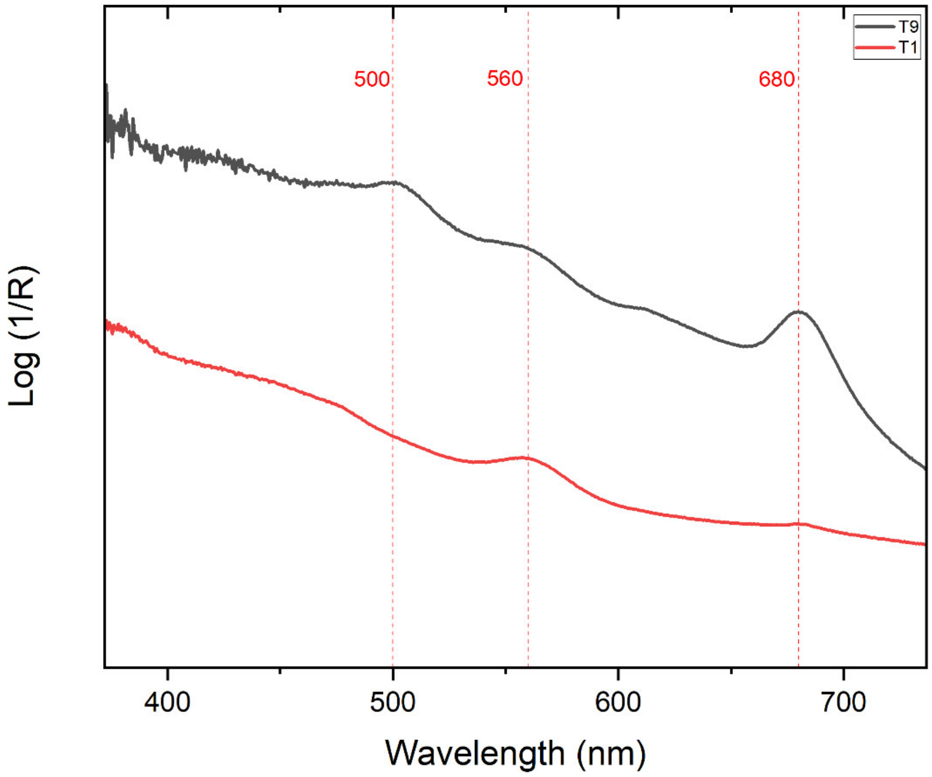

Figure S6: A comparison of the absorption spectra of points T1 and T9.

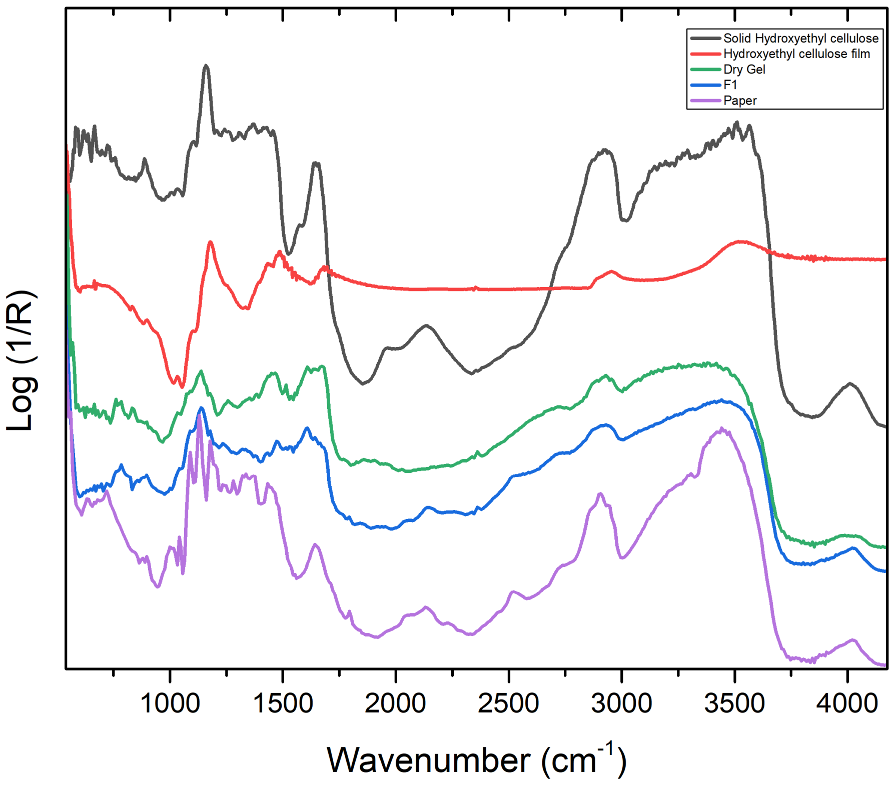

Figure S7: A comparison of FTIR spectra obtained for the mock-up (F1 point), paper, dry-gel and hydroxyethyl cellulose (solid and film from water dispersion).

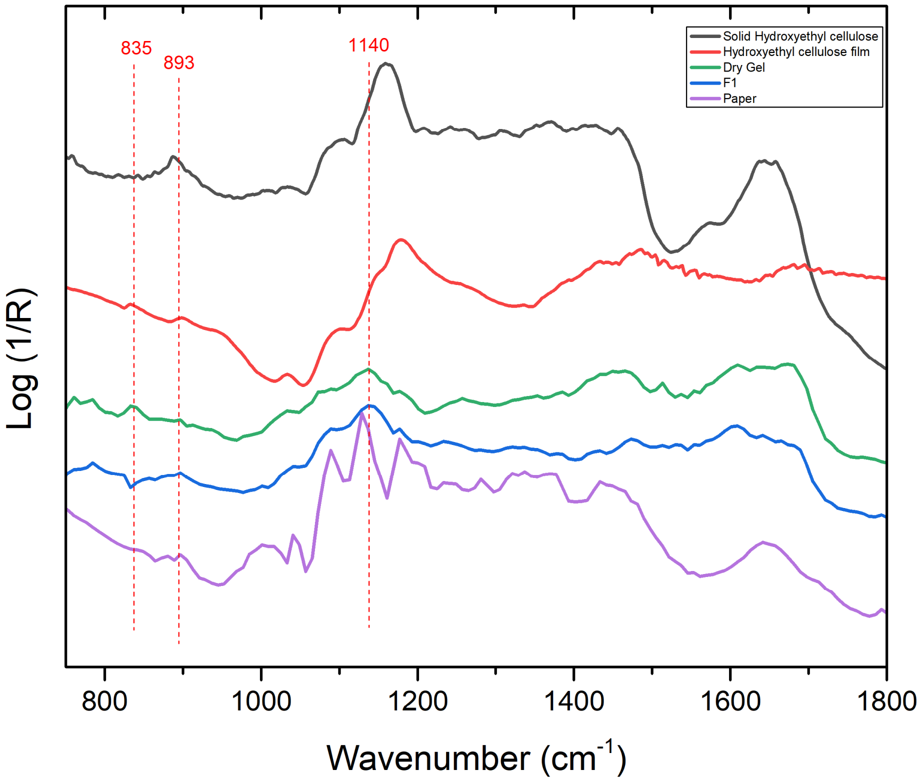

Figure S8: A comparison of FTIR spectra obtained for the mock-up (F1 point), paper, dry-gel and hydroxyethyl cellulose (solid and film from water dispersion) in the range 750-1800 cm^{-1}.

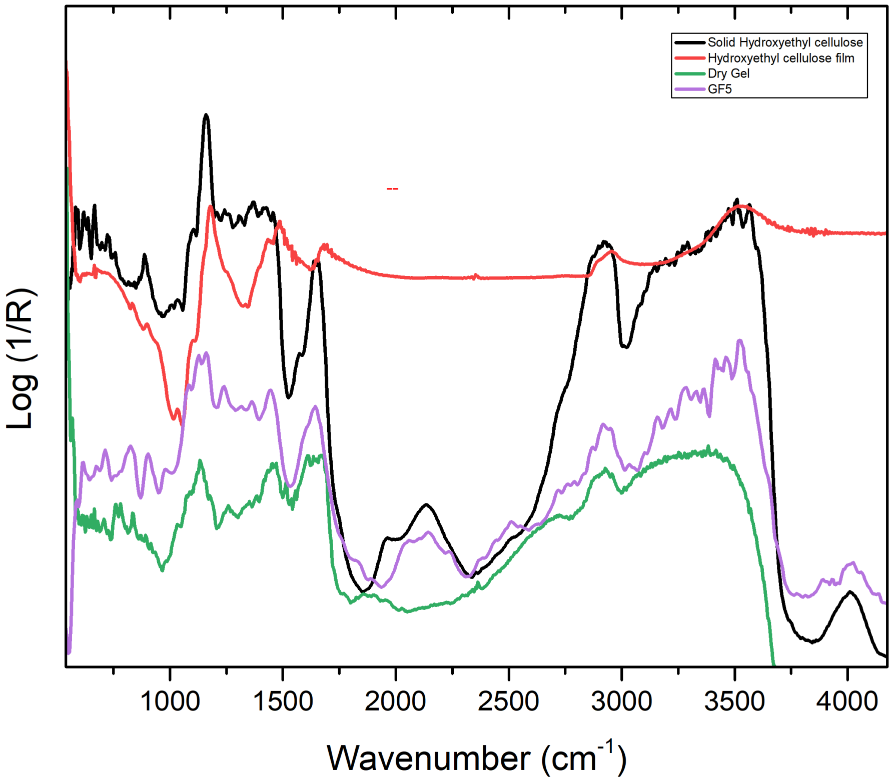

Figure S9: A comparison of FTIR spectra obtained for the artwork (GF5 point), dry-gel and hydroxyethyl cellulose (solid and film from water dispersion).

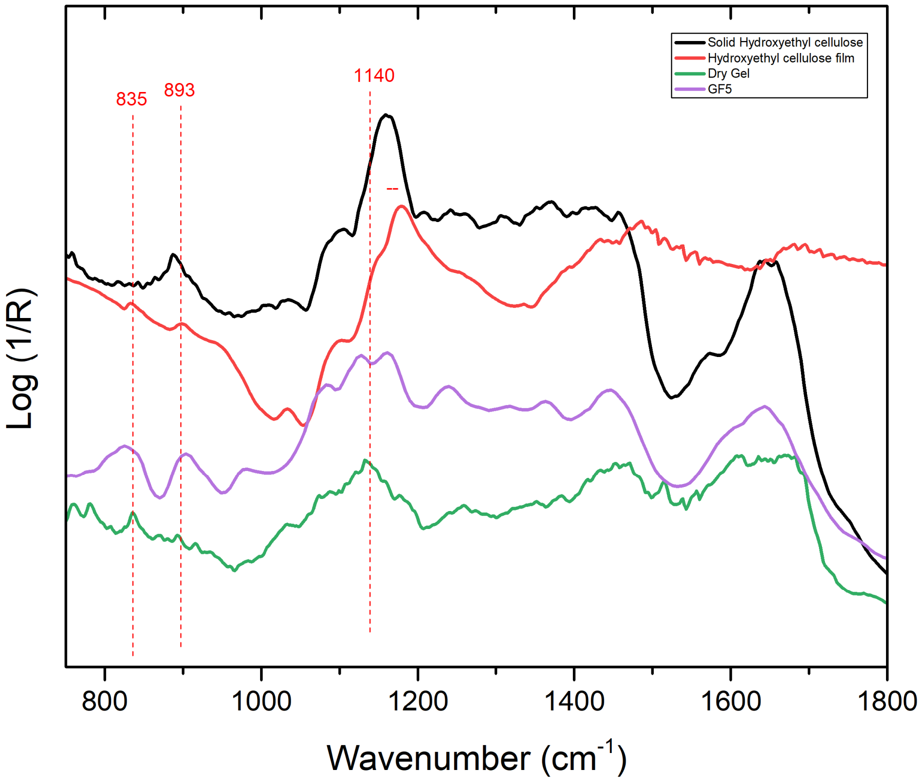

Figure S10: A comparison of FTIR spectra obtained for the artwork (GF5 point), dry-gel and hydroxyethyl cellulose (solid and film from water dispersion) in the range 750-1800 cm^{-1}.
